# Epoxy Composites with Post-Production Gray Cast-Iron Powders

**DOI:** 10.3390/ma17174333

**Published:** 2024-08-31

**Authors:** Robert Cieślak, Paweł Figiel, Konrad Kwiatkowski, Damian Dobrowolski, Magdalena Urbaniak, Anna Biedunkiewicz

**Affiliations:** 1 Department of Materials Technology, Faculty of Mechanical Engineering and Mechatronics, West Pomeranian University of Technology Szczecin, Ave. 19, Piastow, 70-310 Szczecin, Poland; robert.cieslak@zut.edu.pl (R.C.);; 2 Department of Mechanics, Faculty of Mechanical Engineering and Mechatronics, West Pomeranian University of Technology Szczecin, Ave. 19, Piastow, 70-310 Szczecin, Poland; konrad.kwiatkowski@zut.edu.pl (K.K.); magdalena.urbaniak@zut.edu.pl (M.U.)

**Keywords:** epoxy composites, cast iron, waste disposal

## Abstract

Processing of cast-iron castings by machining is associated with a large amount of post-production waste in the form of cast-iron chips, which constitute up to about 5% of the weight of the entire casting. In the case of serial production, this generates large amounts of post-production waste, constituting a constantly growing scale of environmental problems. The aim of this research was to develop a simple and cheap method of utilizing post-production waste of gray cast-iron chips from the machining process for the production of small structural elements of water supply fittings. The analysis of the state of knowledge indicates that the simplest method of managing waste chips is to use them as a starting material in the process of manufacturing polymer composites. The most frequently chosen material for the matrix of polymer composites reinforced with metal powders is epoxy resin. The epoxy composite was produced by the vacuum-assisted casting method. This paper presents the results of tests of morphological, mechanical, and corrosion properties of epoxy composites filled with grey cast-iron powder with a grain size below 0.075 mm and a mass content in the composite of 65%. The composite cured at 130 °C for 90 min had the best mechanical properties. The sample cured at 130 °C for 90 min was observed to have the optimum effect, with a tensile strength of 28.35 MPa, a flexural strength of 55.4 MPa, and a compressive strength of 53.8 MPa. All tested composites were characterized by very good thermal resistance and, in comparison to gray cast iron, over 2.5 times lower weight and an over three times lower corrosion rate in the tap water environment.

## 1. Introduction

The shortage of raw materials is an economic problem because it determines the increase in the costs of both engineering materials and energy. The production of iron castings exceeds 100 million tons in the global economy and is showing an increasing trend [1]. A number of production processes, including cast-iron casting, cutting and milling processes of cast-iron products, such as pipelines, machines, and car parts, elements of mechanical devices, as well as the operation of parts of machines and devices, are sources of emissions of dust, chips, and other forms of waste requiring disposal [2]. Cast-iron shavings are a valuable secondary raw material consisting mainly of iron, with 2.1–4 wt.% carbon and 1–3 wt.% silicon. Machining chips alone usually constitute 3–5% of the casting weight [3]. Much attention has been paid to the issue of such large material loss in the form of chips, necessitating the use of economic recovery and recycling methods, which has been the subject of reports by various researchers for several decades [4,5,6,7,8].

Ground powders are not ready for immediate use. Such powders are largely covered with free graphite particles, which not only complicate the densification process, but also prevent sintering of the parts. Moreover, the presence of existing and emerging oxides, including silicon oxide, during the ball-milling process becomes problematic at both the densification and sintering stages [9,10]. The problem that had to be solved was developing a method of preparing powders for the thickening process. Sintering compacts made of processed iron powder is not an easy task; therefore, the production of sintered materials on an industrial scale is unprofitable [11,12,13,14].

The use of iron powders for the production of friction materials is of great interest. The addition of metallic fibers to these materials has a positive effect on several aspects, including the braking effectiveness of disc brake pads [14,15,16]. Waste materials are being investigated, among others, for applications in civil construction, especially as cement substitutes [16,17,18].

In the production of building materials, the use of iron powders or their combination with cast iron stands out [2,18].

The growing environmental problem of industrial waste can potentially be solved by using it as a filler in polymer composites. The use of composites as metal substitutes is becoming increasingly important in the economy. Compared to steel and alloys of some metals, polymer composites are less susceptible to corrosion and lighter, which results in lower fuel consumption and, therefore, lower operating costs in aerospace and automotive applications. Polymer composites are becoming more and more important in pipeline applications. For centuries, pipes have been constructed from steel, ductile iron, stoneware, bricks, and concrete. These materials react with water and/or sewage, causing corrosion, which affects the failure rate during their operation and shortens their service life. Water leaks that can easily occur due to corrosion can be eliminated by replacing them with new types of plastic pipes or pipes made of composite materials [19]. The appropriate selection of pipe material reduces costs and energy consumption, and at the same time is very important in the context of ensuring the safety and stability of the drinking water supply system and protecting the health of users. A comparison of energy consumption in pipe production processes showed that composite pipes have much lower consumption compared to pipes made of ductile iron, as indicated by the CO_2_ equivalent values [20]. In this respect, composite materials are significantly superior to cast iron, as well as steel and concrete [21]. Polymer composites are characterized by a high strength-to-weight ratio, are resistant to difficult weather conditions and temperature changes, and are better electrical insulators than metals. Their significant advantage is also the possibility of replacing the entire set of metal parts with a single element made of composites, which allows the production of parts with complex shapes. This streamlines the production process and reduces operating costs. Therefore, there is a natural tendency to replace steel and metal alloys with composites in all elements, e.g., from sewage manhole covers to valve covers [22,23]. The role of metal fillers in polymers has been studied by many scientists [21,23,24,25,26,27,28,29,30,31,32,33,34,35,36,37,38,39,40,41,42,43,44,45]. The review of the state of knowledge conducted by Bouzat et al. [43] indicated that the main metals used as fillers in plastic composites are iron, aluminum, copper, zinc, and nickel, whereas the most commonly used polymers are epoxy resins [42,43].

Epoxy resins are thermosetting and/or chemically cured polymers. The advantage of epoxies is their high reactivity. Epoxy resin can withstand temperatures up to several hundred degrees Celsius [46,47].

Epoxy resins are characterized by good mechanical resistance, very low moisture absorption, excellent corrosion resistance, and excellent adhesion to metals [42,48,49]. Additionally, they are less toxic compared to some resins. This commonly utilized composite matrix material is relatively expensive, but its long service life and excellent physical properties often result in a favorable cost-to-performance ratio compared to other polymer materials.

The research results on the production of composites based on epoxy resin with the addition of a mixture of iron carbides or cast-iron powders are presented in [44] and [23,49,50,51,52], respectively.

The state of knowledge based on literature and patent reviews indicates that the only works on the use of gray cast-iron (GCI) chips in the form of an epoxy composite are the works of the authors of the publications. The subject of this research is focused on epoxy composites reinforced with waste gray iron powders for their disposal. The works of Sudheer [23] and Bhavith et al. [49] concern composites reinforced only with waste gray iron powders in an epoxy resin matrix. In the work of Chung et al. [51], the effect of gray iron powder addition on the rheological and mechanical properties of an epoxy composite reinforced with aluminum powders and short fibers manufactured using a technique belonging to the Rapid Prototyping technology family was studied. The addition of cast-iron powders increased the tensile strength from 41 to 72 MPa compared to the resin, and from 31 to 72 MPa compared to the composite without cast-iron powder. The effect of gray iron powder addition on the damping properties of an epoxy composite reinforced with granite powders was also studied in [50]. Vibration test results of the granite epoxy composite with the addition of grey cast-iron powder showed a significant increase in the damping properties of the epoxy composite. Chips obtained during the machining of cast iron were pre-cleaned and heated to remove the moisture/oil content [49]. The next step was the grinding processes using a hammer mill and the selection of a powder with a size of 75 µm. The matrix was a thermosetting epoxy resin (LY556). Increasing the cast-iron content from 0.5 to 25 wt.% was promoted to increase the hardness of epoxy composites. The porosity of the obtained composites contributed to the decrease in fatigue resistance, water penetration, and weather resistance. The increase in the cast-iron content reduced the bending strength, but improved the tensile properties, hardness, impact strength, and density of the composites. The best results were obtained for the composite with the highest packing density, i.e., 25 wt.% of cast-iron filler. For the composite, the tensile strength and tensile modulus were 68.74 MPa and 4.01 GPa, respectively, and for the resin, 52.42 MPa and 3.37 GPa. The flexural strength and flexural modulus for the composite were 65.228 MPa and 92.881 GPa, respectively, and for the resin, 92.882 MPa and 5.17 GPa. In the opinion of the authors of this paper, the epoxy composite with 25 wt.% of cast-iron filler has potential in low-load applications, such as car door panels, table tops, and furniture.

Sudheer et al. [23] presented the method of preparing gray iron powders in the process of manufacturing epoxy composites. Preparation of gray iron powders was carried out in a different order, i.e., first, cast-iron powders collected from the mechanical workshop were sorted to obtain fine-grained powders (≤50 µm) and then heated at 400 °C in a muffle furnace for two hours. Composites with 10 and 20 wt.% powder content were prepared using the casting procedure, maintaining the suspension in a state of continuous mixing. The subject of the study were composites with 0, 10, and 20 wt.% powder content in a thermosetting epoxy resin type LY556. The addition of gray cast-iron powder resulted in the improvement of the epoxy resin properties and increased density and hardness. Rockwell’s hardness of the composite with 20 wt.% powder addition was 83. A positive effect of the filler on friction and epoxy wear was found.

Compared to the above-described methods of manufacturing epoxy composites filled with grey cast-iron powder, this paper presents a simple method of chip preparation, consisting of cleaning and degreasing post-production chips from grey cast iron in technical acetone. Chips and powders are not subjected to heat treatment, which reduces the costs of the technology. After crushing and sieving, no procedures were used to eliminate free carbon. The added value of the developed technology is the degassing and homogenization of powders in a mixture of epoxy resin and curing agent assisted by vacuum, and then rapid cross-linking of the homogeneous mixture after just 3 s at a temperature of 130 °C, which is presented in the following sections. The hitherto unattained 65 wt.% percentage share of grey cast-iron powders in epoxy resin reduces the share of resin needed for disposal, increases the efficiency of the utilization process of post-production waste of grey cast-iron chips, and thus reduces the costs of managing environmentally harmful waste and losses incurred by manufacturers of cast-iron structural elements. These composites could be a very good candidate for the production of small structural elements of water supply fittings, such as covers, hydrant valve boxes, sewer elbows, connectors, sleeves, and others.

## 2. Materials and Methods

### 2.1. Materials and Processing

A commercial bisphenol-A-based epoxy resin and a compatible epoxyaminoamine cross-linker were used for research purposes. The following reagents and colorimetric indicators were employed: glacial acetic acid and crystal violet, purchased from Chempur (Piekary Slaskie, Poland), chloroform from P.P.H. Stanlab (Lublin, Poland), tetraethylammonium bromide, provided by Acros Organics (Geel, Belgium), and perchloric acid standard solution 0.1 M in glacial acetic acid, supplied by Fischer Chemicals (Zurich, Switzerland). All chemicals were of analytical grade and were utilized without further purification.

The starting material for the filler of epoxy resin/gray cast-iron composites was post-production cast-iron chips from machining delivered by WODROL (Wałcz, Poland). The mechanical and strength parameters with the chemical composition of cast iron are summarized in Table 1.

**Figure 1:**
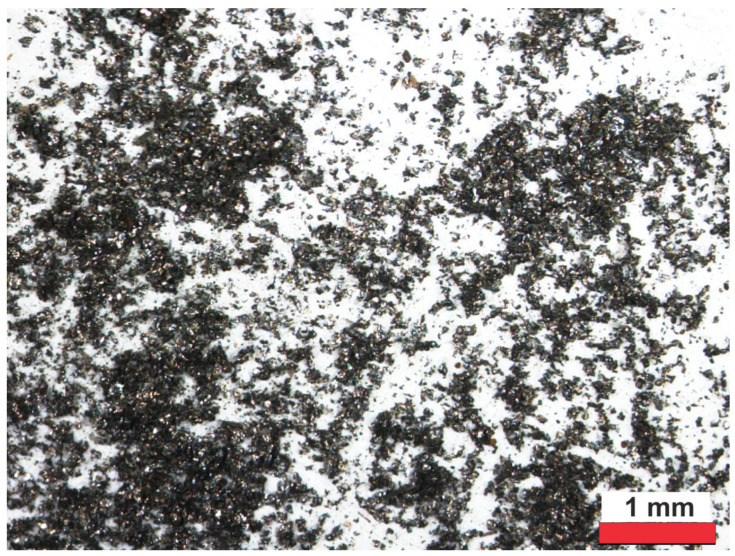
Microscopic image of cast-iron fraction powders with grain size below 0.075 mm.

For testing, gray cast-iron chips were cleaned and degreased in technical acetone from OFO sp. z o.o. (Piechocin, Poland). In the next stage, they were ground in ethyl alcohol using a Pulverisette 4 planetary grinder from Fritsch GmbH (Idar-Oberstein, Germany). The ratio of the mass of the balls to the mass of the chips was 5:1. The grinding speed was 200 rpm, and the grinding time was 3 h in cycles of 3 × 45 min, with breaks of 15 min. The powder was dried in the air. The powder prepared in this way was subjected to sieve analysis and separated into seven fractions using the LPZE-1 sieve analyzer. The presence of free carbon was observed in each of the seven fractions of the prepared gray cast-iron powder. The fraction of gray cast-iron powders with the smallest grain sizes < 0.075 mm was selected for further research (Figure 1).

The initial assumptions for the project included the highest possible cast-iron content in the composite. Preliminary laboratory tests have shown that the maximum possible mass fraction of gray cast-iron powders with a grain size smaller than 0.075 mm is 65%. In this assessment, the criterion of accurate filling of the casting mold was used. The composition of the resin-to-curing agent ratio was in accordance with the manufacturer’s recommendations. The components were mixed thoroughly until a homogenous mixture was achieved. The resin, curing agent, and gray cast-iron powders were mixed under a reduced pressure of 0.01 MPa using a dispersing device until a degassed and homogeneous suspension was obtained. The size of the prepared portion of the suspension determined the duration of this stage. The prepared mixture was filled into casting molds made of Polyamide PA6 by P.H.U. SZCZEL-PLAST S.C. (Katowice, Poland) under room-temperature (RT) conditions.

**Figure 2:**
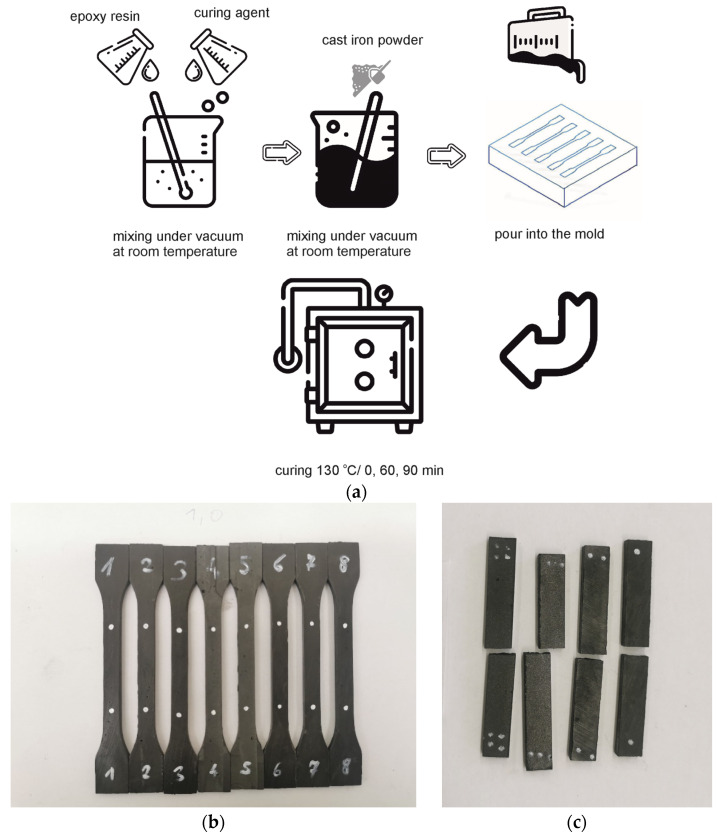
Flowchart of the sample preparation procedure (**a**) and images of GCI/epoxy-based composite: (**b**) prepared for static tensile tests and (**c**) after static flexural tests.

In the next step, the liquid mixture was heat-treated. It was assumed that in order to harden the GCI/epoxy-based composite, it would be beneficial to heat the mixture at a temperature higher than the cross-linking temperature recommended by the epoxy resin manufacturer. This favors the material structure’s reorganization, lowers the system’s viscosity, and thus improves the consolidation conditions while shortening the cross-linking time of the composite. After heating, the mold was cooled at ambient conditions. The manufacturing procedure, with images of samples prepared for testing after casting into a mold, heating, and preparation for static tensile tests and composite samples after static flexural tests, is shown on Figure 2.

The composite samples were polished with P150 sandpaper and then milled to achieve a thickness in accordance with the standard. For morphological tests and hardness measurements, the composite samples were grinded with P120 and then P500–P1200 sandpaper.

### 2.2. Methods

#### 2.2.1. Characteristics of Gray Cast-Iron Powders

Digital microscope VHX-7000 (Keyence, Osaka, Japan) series were used to examine the cast-iron powders (Figure 1). The gray cast-iron powders were identified by the X-ray diffraction method. The PW3040/60 X’Pert Pro apparatus (Malvern Panalytical Ltd., Malvern, UK) equipped with Cu Kα radiation was used. The X’PertHighScore 1.0 software with a built-in ICDD spectra library (ICDD database) was used to process and analyze the XRD spectra.

A comparative analysis of the free carbon content in the cast-iron powder was conducted using thermo-analytical techniques with a TG-DSC apparatus (SDT Q600, TA Instruments, New Castle, DE, USA) coupled with a mass spectrometer (Thermostar GDS 301 Pfeiffer Vacuum, Aßlar, Germany) for gaseous products’ identification. The last three ultrafine fractions of gray cast-iron powder with grain sizes in the ranges of 0.102–0.088 mm, 0.088–0.075 mm, and less than 0.075 mm were selected for testing the heating process in dry air. Measurements were performed under non-isothermal (β = 10 K/min) conditions, over a temperature range of 25 to 1250 °C. Throughout the process, the sample temperature, weight change (TG), heat flow (HF), and CO_2_ ion signal were recorded over time.

#### 2.2.2. Characterization of Matrix Material Components and Performance Evaluation

The viscosity tests were carried out using the AMETEK Brookfield DV2T Viscometer at room temperature (spindle 3, speed 20 RPM). The non-volatile-matter content (NV) of epoxy resin was evaluated thermogravimetrically, using a moisture analyzer, MAX 60/NP (Radwag, Radom, Poland), according to ISO 3251:2019 standard [53]. Analysis was performed at 140 °C for a duration of 30 min. The sample weight was approximately 1 g. NV (%) = (m_2_/m_1_) × 100%, where m_1_ is the weight of the epoxy resin sample, and m_2_ is the residual weight of the sample after heating.

Epoxy equivalent (EE) was determined by means of colorimetric titration, based on the methodology described in ISO 3001:1999 standard [54]. Briefly, epoxy resin samples were dissolved in chloroform (10 mL) and combined with 20 mL of 0.1 N glacial acetic acid and 10 mL of tetraethylammonium bromide solution prior to titration. The volumetric analysis was carried out with the standard solution of perchloric acid solution (0.1 N) to achieve a stable green color in the presence of crystal violet indicator (titration endpoint). EE (g/mol) was calculated using the following equation:

$$EE=\frac{1000\;m}{\left(V_{1}-V_{0}\right)\left(1-\frac{t-t_{s}}{1000}\right)c}$$

where: *m*—epoxy resin sample weight (g), *V*_0_—volume of the perchloric acid solution used during the control experiment (mL), *V*_1_—volume of the 0.1 N perchloric acid utilized in the titration (mL), *t*—temperature of the perchloric acid during both the *EE* and blank tests (°C), *t_s_*—temperature of the perchloric acid solution during the titer adjustment (°C), and *c*—concentration of perchloric acid solution (mol/L).

The thermal properties of epoxy composition were studied by differential scanning calorimetry (DSC) using the Netzsch DSC 204 Fhoenix (Selb, Germany) instrument, calibrated against indium and n-octane standards. The measurement was carried out for 10 mg samples in a nitrogen atmosphere using a standard 40 μL aluminum crucible. The DSC testing cycle consisted of heating–cooling–heating scans, with the heating/cooling rate of 10 °C/min, from −50 to 250 °C.

The infrared spectra were acquired with a Thermo Nicolet 380 FTIR spectrometer working in Attenuated Total Reflectance mode (ATR-FTIR). Here, 64 scans at a resolution of 4 cm^−1^ were averaged for each sample in the range of 4000–400 cm^−1^ to record the spectra. The chemical composition of the resin and curing agent were identified by ^1^H NMR spectroscopy studies. The ^1^H NMR spectra were recorded in CDCl_3_ containing 0.03% (*v*/*v*) TMS (tetramethylsilane) on a BRUKER DPX-400 Avance III HD spectrometer (Billerica, MA, USA) operating at 400.13 MHz. Since curing behavior with respect to temperature is essential to control the thermoset processing, the FTIR curing characteristics of the resin were studied at the temperature employed further in composite manufacturing, whilst the curing time was fixed at 80 min.

#### 2.2.3. Microstructural Characterization of the GCI/Epoxy-Based Composites

To assess the morphology and microstructure of the composite, the FE-SEM SU-70 (field emission scanning electron microscopy) microscope (Hitachi, Naka, Japan), equipped with a microanalysis adapter EDS (energy dispersive spectrometry) from Thermo Fisher Scientific (Madison, WI, USA), was used. During scanning electron microscopy examinations, the backscattered electron (BSE) and the secondary electron (SE) were registered. The density of epoxy resin and GCI/epoxy-based composites was determined using the Archimedes method.

#### 2.2.4. Mechanical Properties

Shore D hardness was determined using the Durometer Zwick 3100 (Zwick AG, Germany). For each sample, the measurement was carried out 10 times, each time in a different place of the sample, and the final result was the average with the calculated confidence interval in accordance with the ISO 2602:1980 standard [55].

Strength tests were carried out using the INSTRON ElectroPuls E10000 (Norwood, MA, USA) testing machine. The tests included a static tensile test carried out at a speed of 5 mm/min, in accordance with the ISO 527-1:2019 and ISO 527-2:2012 standards [56,57]. A static compression test was carried out at a speed of 2 mm/min in accordance with the ISO 604:2002 standard [58]. The static flexural test was carried out at a speed of 2 mm/min in accordance with the ISO 178:2019 standard [59]. A hammer with an energy of 2 J was used for the Charpy impact test.

#### 2.2.5. Corrosion Testing

Samples with a diameter of 14 mm and a thickness of 8 mm were prepared for corrosion tests in a three-electrode system. The polarization curves of the samples were measured in an electrolytic cell containing approximately 1000 mL of tap water with natural oxygen under RT conditions. The Atlas 9833 Electrochemical Interface Potentiostat from Atlas-Sollich (Rebiechowo, Poland), controlled from a computer using the Pol99-win v. 1.4 software, was used for the measurements. The tested electrode was a sample with an area of 1 cm^2^. A calomel electrode with a potential of 0.244 V (at 25 °C) placed in the Ługin capillary was used as the reference electrode. This capillary connects the reference electrode with the near-surface area of the tested sample. It serves to reduce the ohmic polarization that occurs in the electrolyte itself. The auxiliary electrode was a graphite electrode. Corrosion tests were performed in the polarization range from −2.0 to + 2.0 V at a scanning rate of 1 mV/s. The Tafel Slope extrapolation method was applied to determine the corrosion parameters: corrosion potential (E_cor_), corrosion current (i_cor_), and linear corrosion rate (CR), using the AtlasLab v. 2.20 software.

#### 2.2.6. Testing the Thermal Resistance

A comparative assessment of the thermal resistance of the GCI/epoxy-based composites and a reference sample, such as epoxy resin, was carried out using the thermo-analytical (DSC) method under nitrogen atmosphere conditions. The thermal decomposition process of epoxy resin revealed a convex peak on the DSC curve, indicating an increase in enthalpy (exothermic process).

## 3. Results

### 3.1. Characterization and Performance Evaluation

#### 3.1.1. Characterization of the Gray Cast-Iron Powder

**Figure 3:**
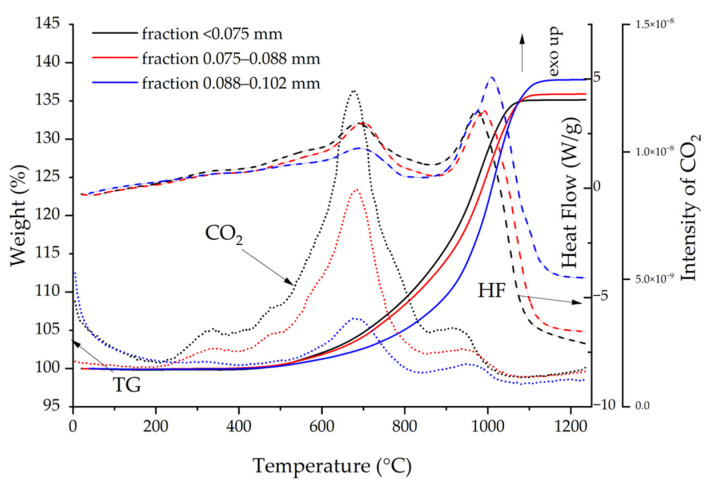
TG-DSC-MS analysis of cast-iron powders. Plots of the TG, HF, and mass spectra of CO_2_ obtained when heating three fractions of gray cast-iron powders in dry air.

Three ultrafine fractions of gray cast-iron powder with grain sizes ranging from 0.102 to 0.088, 0.088 to 0.075, and <0.075 mm were selected for the assessment of free carbon content using the TG-DSC-MS method. Figure 3 shows the recorded TG, HF curves, and mass spectra of CO_2_ released, as a function of temperature.

Figure 3 shows the total test results of the last three fractions of the analyzed powder with grain sizes of 0.102. It was 0.088, 0.088–0.075, and <0.075 mm, respectively. Estimated calculations showed that the powder fraction with grain size <0.075 mm contained approximately 1.5 mass% of elemental carbon more than the fraction in the range of 0.102–0.088 mm. At temperatures below 800 °C, elemental carbon burned, and at temperatures above 900 °C, carbon bound in the cast-iron structure in the form of carbides burned [60]. These reactions were accompanied by exothermic effects visible in the HF curves (dash lines). The comparison of TG curves (solid lines) and CO_2_ mass spectrometry signal (dot lines) intensities indicated that the fraction with the smallest grains below 0.075 mm contained the highest content of free carbon separated from the cast-iron flakes during the grinding process, and the largest share of unbound carbon increased with the decreasing grain size.

**Figure 4:**
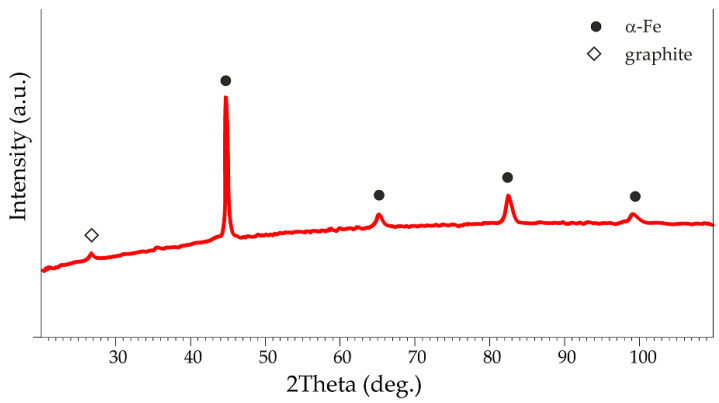
The X-ray diffraction pattern of the gray cast-iron chips.

Figure 4 shows the diffractogram of gray cast-iron chips. The analysis of its phase composition allowed the identification of two phases typical of gray cast iron, i.e., α-ferrite (ICDD Card No. 00-006-0696) and graphite (ICDD Card No. 00-025-0284).

#### 3.1.2. Characteristics of an Epoxy System

Table 2 shows the results of testing the viscosity at room temperature (η), the content of non-volatile substances (NV), and the epoxy equivalent (EE) of the epoxy resin.

The obtained data suggested that we were dealing with a resin that has a low viscosity (920 cP at RT), a relatively significant amount of volatile matter content (over 24 wt. %), and a notable concentration of epoxy groups (EE = 192.38 g/mol).

#### 3.1.3. Characteristics of an Epoxy Resin and a Curing Agent

**Figure 5:**
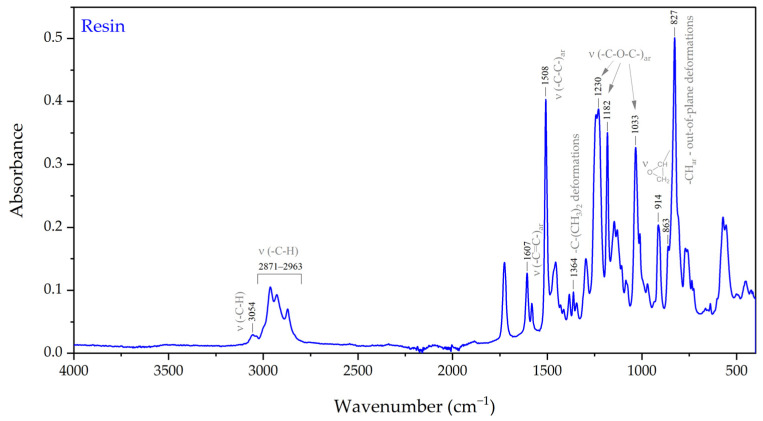
FTIR spectrum of uncured epoxy resin.

**Figure 6:**
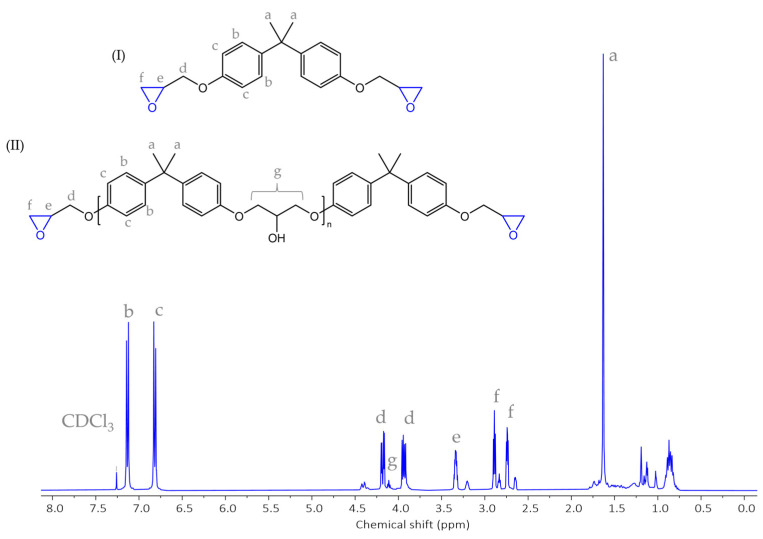
The ^1^H NMR spectra of epoxy resin (I—DGEBA and II—DGEBA oligomers).

In order to verify the molecular structure of the individual components of the epoxy resin system, a set of ATR-FTIR and ^1^H NMR spectroscopic investigations was conducted. The ATR-FTIR spectra of the epoxy resin and curing agent are shown in Figure 5 and Figure 6, respectively. The spectra of the epoxy resin showed features characteristic of epoxy systems with bands resulting from the C-O stretching vibrations of the oxirane group at 863 and 914 cm^−1^ and the stretching vibrations of the terminal -C-H group in the epoxy ring at 3054 cm^−1^. Moreover, a number of bands corresponding to the symmetrical and asymmetrical stretching vibrations in ether linkages, ν(C-O), could be easily distinguished at 1033, 1182, and 1230 cm^−1^. Additionally, bands characteristic for C-C and C=C stretching vibrations and aromatic C-H out-of-plane deformations in aromatic benzenic rings appeared around 1508, 1607, and 827 cm^−1^, respectively. Several weak bands at 2871–2963 cm^−1^ corresponded to symmetrical and asymmetrical C-H stretching vibrations in CH_2_ and CH_3_ groups, and the band at around 1364 cm^−1^ corresponded to C-(CH_3_)_2_ deformation vibrations [61,62,63]. Based on the above, we can assume that bisphenol-A-diglicydyl ether (DGEBA)/bisphenol-A-based epoxy resin is a key component of the investigated system.

The main features of the resin and ^1^H NMR spectra were found to be in good agreement with the FTIR spectroscopic data. As shown in Figure 6, signals corresponding to the resonance of aromatic protons of bisphenol-A (BPA) units exhibited doublets at δ = 7.14 ppm (b signal) and δ = 6.8 ppm (c signal), whilst the methyl protons yielded a singlet at δ=1.65 ppm (signal a). The group of resonances in the region, 3.8–4.2 ppm, 3.3 ppm, and 2.70–2.90 ppm, corresponded to glycidyl terminal group protons (d, e, and f signals) [64,65]. Moreover, we also observed the resonance signal at δ = 4.10 ppm, which can be attributed to the aliphatic chain protons (signal g), identified by some authors as a special feature of epoxy resin oligomers characterized by a degree of polymerization greater than zero (n > 0) [66]. These results strongly suggest that epoxy resin should be regarded as a mixture of DGEBA (Figure 6, structure I) and its oligomers (Figure 6, structure II).

**Figure 7:**
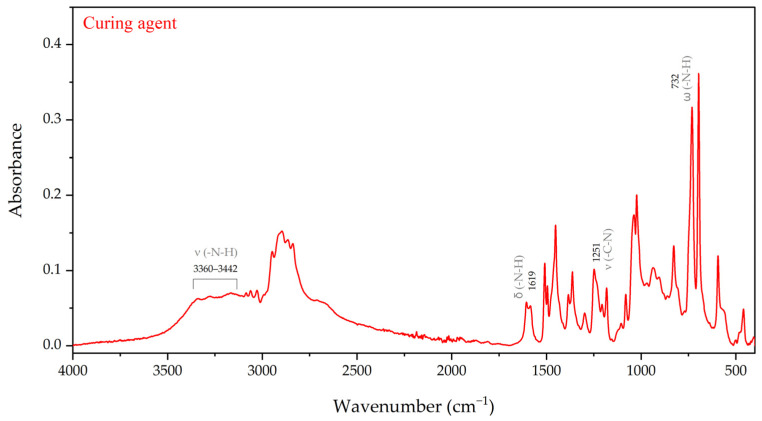
FTIR spectrum of the epoxy curing agent.

The FTIR spectrum corresponding to the curing agent is represented in Figure 7. It can be confirmed that we are dealing with an amine-based curing agent, as the bands associated with vibrations of amine groups are distributed along the whole spectrum range. The sharp absorption bands at 732, 1251, and 1619 cm^−1^ in the curing agent spectrum can be attributed to -N-H wagging, -C-N stretching, and -N-H bending vibrations, respectively. Moreover, some less intense peaks due to -N-H stretching vibrations were also evident at 3360–3442 cm^−1^.

**Figure 8:**
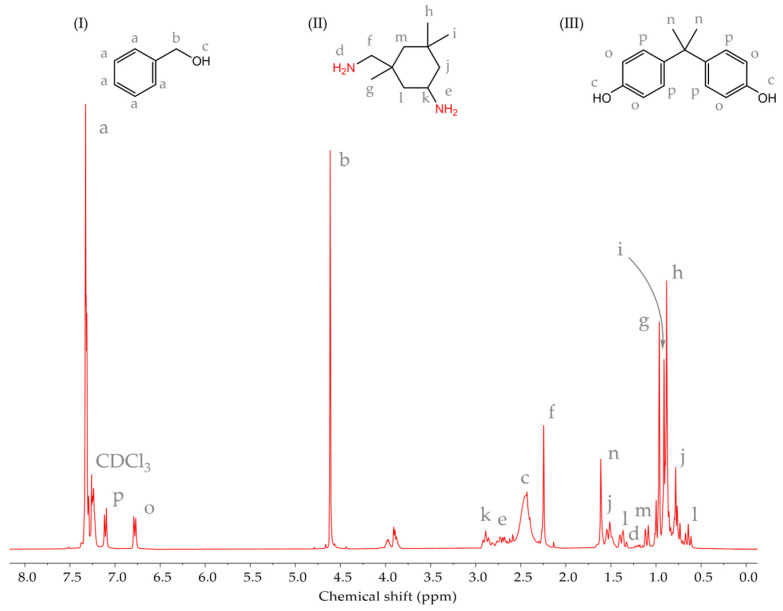
The ^1^H NMR spectra of the curing agent (I—benzyl alcohol, II—IPDA, and III—BPA).

The chemical structure of the curing agent was confirmed through ^1^H NMR investigations. Based on the registered ^1^H NMR spectra (Figure 8), the compound in question is based on 3-aminomethyl-3,5,5-trimethylcyclohexylamine, widely known as isophoronediamine (IPDA)—a cycloaliphatic diamine curing agent characterized by low viscosity and rather moderate reactivity. In the NMR spectrum of the curing agent, characteristic singlets corresponding to the IPDA methyl protons (-CH_3_) appeared at 0.8–0.9 ppm (signals h, i, and g). Moreover, several signals due to methylene protons in the cycloaliphatic ring and bridging -CH_2_- groups could be observed at 1.5 ppm (signal j), 1.4 ppm (signal l), 1.0 ppm (signal m), and 2.3 ppm (signal f), whilst those corresponding to the methine protons occurred as a multiplet at 2.9 ppm (signal k). Besides, resonance signals attributable to the protons of an aliphatic- and a cycloaliphatic-type (e signal) amine group could be distinguished at 1.2 ppm (d signal) and 2.7 ppm (e signal), respectively. These peak assignments aligned closely with the findings reported earlier by Kárpáti et al. [67]. Apart from the main component, the ^1^H NMR studies of the amine curing system revealed the presence of an organic solvent, i.e., benzyl alcohol. The characteristic proton resonance signals at 7.3 ppm representing the aromatic -CH_2_- (signal a), 4.6 ppm due to aliphatic methylene protons (signal b), and 2.5 ppm due to -OH protons (signal c) were consistent with those expected for benzyl alcohol. At this point, it is worth mentioning that the incorporation of a hydroxyl-containing solvent into a thermosetting system is a common commercial practice to reduce the system’s viscosity [68]. Lastly, as evidenced by the presence of aromatic proton resonance signals at 6.8 ppm (signal o) and 7.1 ppm (signal p), the singlet at 1.5 ppm attributed to -CH_3_ groups (signal n), and the signal at 2.5 ppm due to the presence of -OH protons (signal c), a minor quantity of BPA could be detected in the curing system under investigation.

#### 3.1.4. Cure Monitoring and Characterization

**Figure 9:**
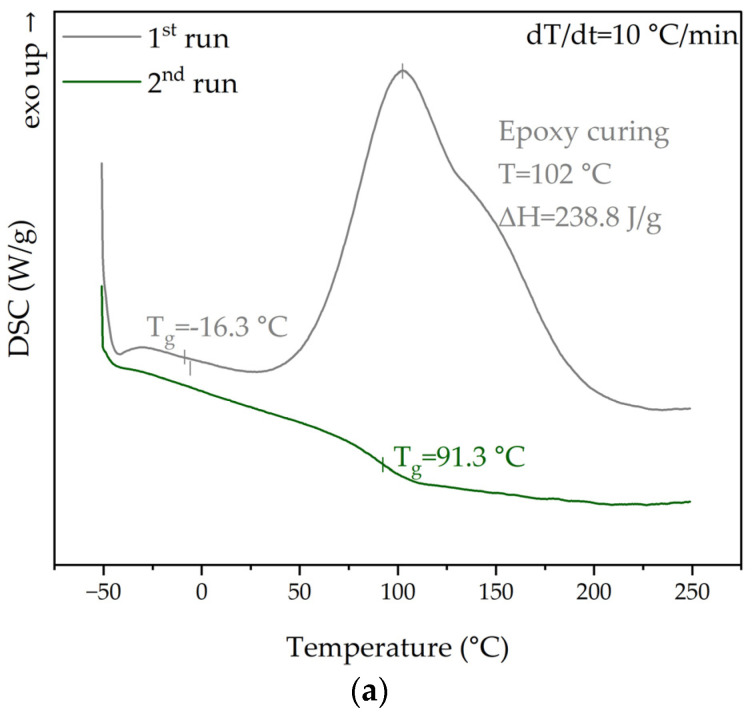

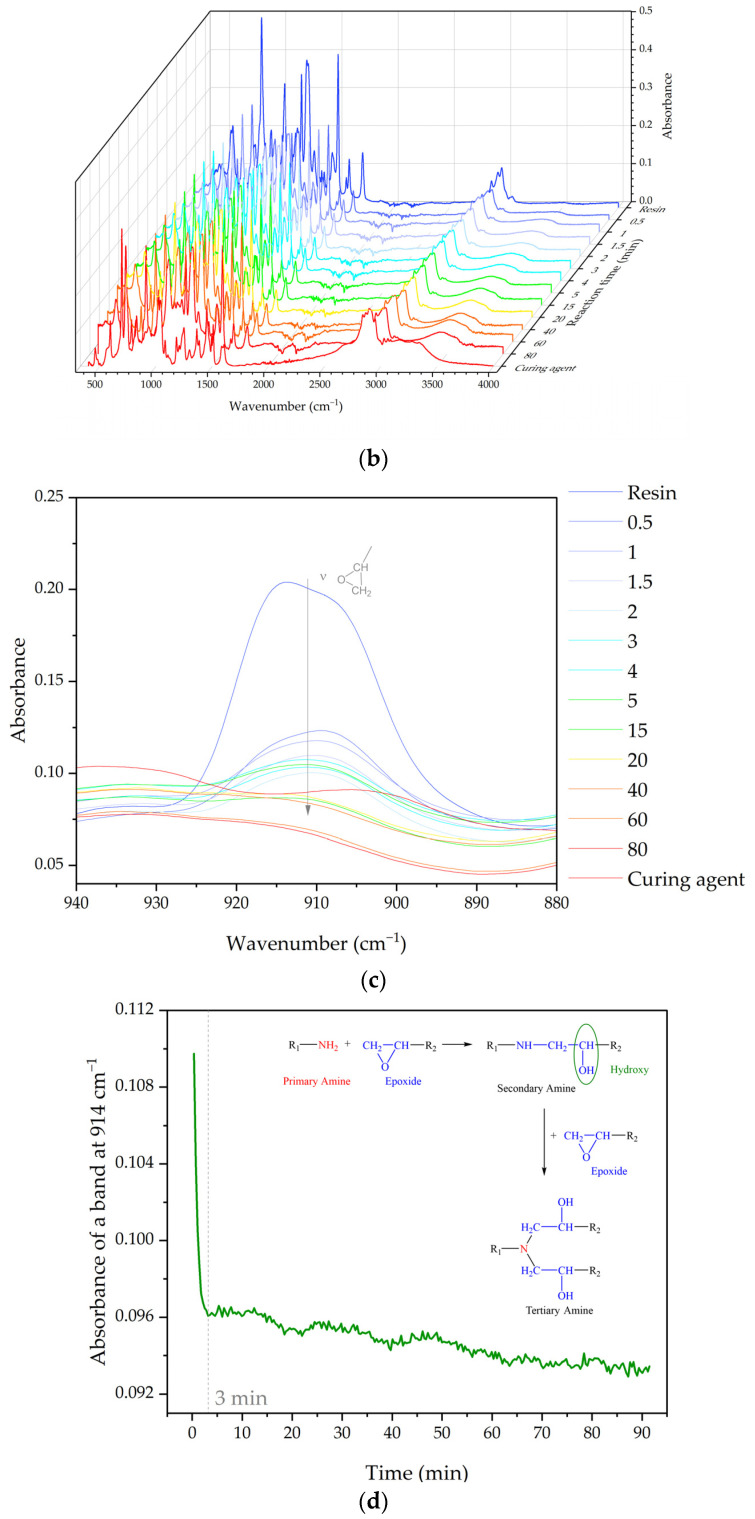
DSC scans comparing both uncured (1st scan) and cured samples (2nd scan) (**a**). Curing monitoring—FTIR spectra of resin, curing agent, and the reaction product between them (**b**), changes in absorbance intensity of the band representing the C-O stretching vibrations of oxirane group at 914 cm^−1^ (**c**), and absorbance–curing time profile together with the general reaction mechanism between epoxy and amine curing agents (**d**).

Figure 9a shows the DSC results of the uncured and cured epoxy system, with 1st and 2nd DSC heating scans, respectively. It can be seen that the uncured sample exhibited a single glass transition in the low-temperature range (Tg = −16 °C) and an exothermic curing reaction peak at a temperature of 102 °C, characterized by a total heat of 238.8 J/g. Based on the pattern of the exothermic peak, we deduced that the curing initiation temperature was ~50 °C, whilst the process was fastest at a temperature >100 °C (peak temperature). In the second run, the calorimetric curve displayed a single inflection point corresponding to the glass transition temperature (Tg = 91.3 °C), without any detectable heat of the curing reaction. In order to gain some insight into the reaction course, the band representing the C-O stretching vibrations of the oxirane group at 914 cm^−1^ was utilized to monitor the progress of curing over the reaction time (Figure 9b–d). The initial spectra of both the resin and curing agent were consistent with those recorded earlier. The introduction of the curing agent after 30 s already indicated significant changes in the FTIR spectrum, specifically a decrease in the intensity of the band arising from the C-O stretching vibrations of the oxirane group at wavenumber 914 cm^−1^. A significant reduction in the intensity of this band was noticed after just 30 s, and this reduction became more pronounced as the curing reaction progressed (as clearly observed in the enlarged spectrum view in Figure 9c). It was found that, within the given conditions, the reaction proceeded very quickly during the first 3 min, and approximately 70 min were required for complete curing, as indicated by a plateau on the absorbance–curing time profile (Figure 9d). Such changes, marked by a decrease in the intensity of the band related to epoxy group vibrations, were associated with the consumption of epoxy groups (epoxide ring-opening) reacting with a primary amine. By examining the reaction scheme shown in Figure 9d, we found that as a result of the curing reaction, new hydroxyl groups were formed, as confirmed by the occurrence of a broadband corresponding to -OH groups’ stretching vibrations at 3450 cm^−1^ (Figure 9b).

### 3.2. Conditions of the Heat Treatment Process of GCI/Epoxy-Based Composites

The heat treatment of the suspension containing the resin, gray cast-iron powder, and curing agent was carried out at a temperature approximately 45 degrees higher than the cross-linking temperature recommended by the epoxy resin manufacturer.

The subject of the research was three types of GCI/epoxy-based composites differing in the conditions of the final stage:

✓ heated to 130 °C, not soaked, samples marked with the symbol Pr65 2/0,
✓ heated at a temperature of 130 °C for 60 min, samples marked with the symbol Pr65 2/1,
✓ heated at a temperature of 130 °C for 90 min, samples marked with the symbol Pr65 2/1.5.

### 3.3. Microstructure of the GCI/Epoxy-Based Composites

**Figure 10:**
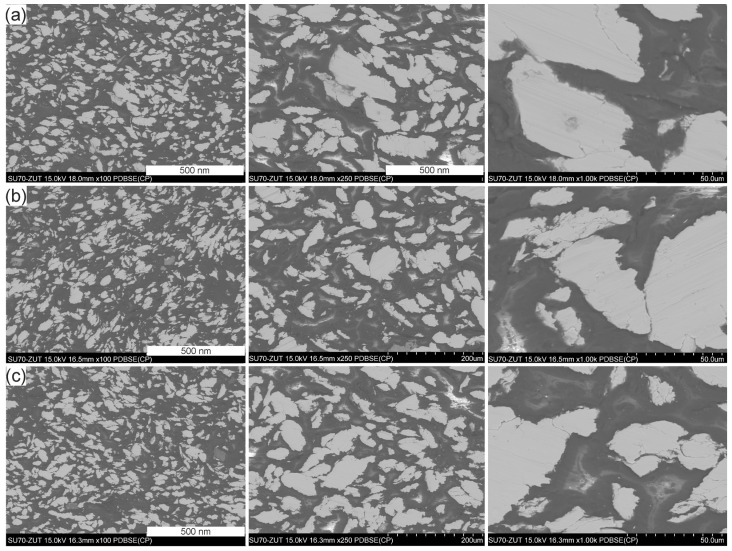
BSE microstructure images of the cross-section of the Pr65 2/1 (**a**), Pr65 2/1.5 (**b**), and Pr65 2/0 (**c**) composites.

**Figure 11:**
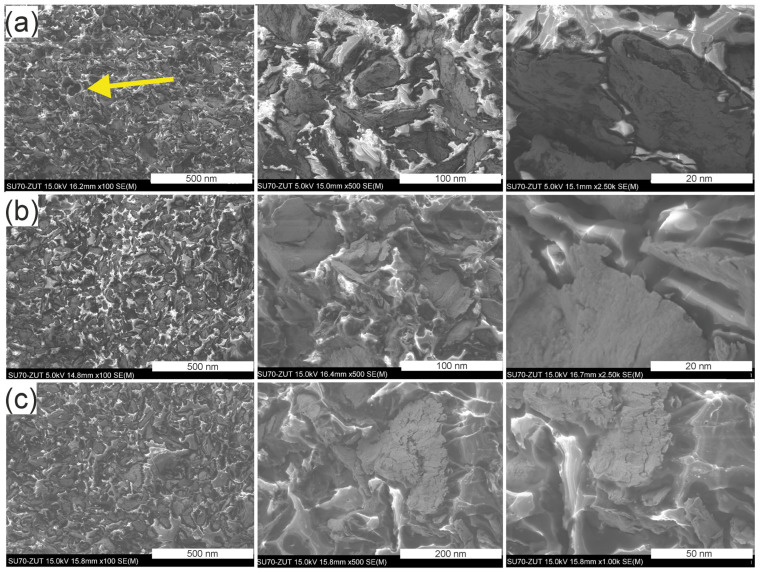
Scanning electron microscopy images of the fracture surfaces of samples Pr65 2/1 (**a**), Pr65 2/1.5 (**b**), and Pr65 2/0 (**c**) after the tensile test.

Figure 10a–c shows scanning electron microscope images of the cross-sectional surfaces, while Figure 11a–c show the fracture surfaces of the tested GCI/epoxy-based composites.

An analysis of images of the microstructure of the GCI/epoxy-based composites showed that the particles were dispersed and distributed homogenously in the epoxy matrix (Figure 10a–c). A rougher surface of the powder-reinforced epoxy resins was found on the fracture surfaces of the composites. Symptoms of brittle fracture of composites can be observed after tensile tests. The failure mechanism of the composite is manifested by cracking of the matrix, and the formation of a phase boundary was observed at the interface with weak interaction of the metal filler with the polymer matrix, as evidenced by partial separation of particles from the matrix. The formation of a phase boundary with a weak interaction between the metal filler and the polymer matrix was observed at the phase boundary, as evidenced by the partial debonding of the particles from the matrix (Figure 11a–c). This could be explained by the presence of air voids in the composites. However, no loss of reinforcement particles from the matrix was observed after tensile tests. Only accidental particle releases were found, as indicated by arrow a in Figure 11a. At the interface, adhesion of the matrix with gray cast-iron powders was ensured by mechanical anchoring of the matrix in the significantly developed surface of the metallic filler, which also positively contributed to the effect of Van der Waals forces. This hypothesis requires further research to clarify it.

**Figure 12:**
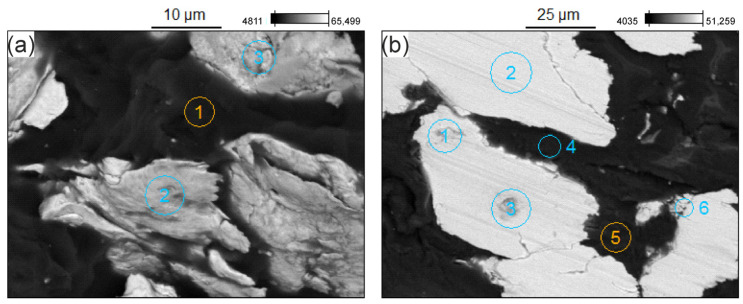
EDS images of the Pr65 2/1.5 composite: fracture (**a**) and grinded cross section surface (**b**).

Example results of the microanalysis of the chemical composition of the Pr65 2/1.5 composite obtained using the energy dispersive X-ray spectrometer (EDS) technique are presented below in Figure 12a,b. Point 1 on the SEM micrographs (Figure 12a) corresponds to the resin (C and O), and points 2 and 3 to cast iron (Fe and Si). Points 4 and 5 on the SEM micrographs (Figure 12b) correspond to resin (C and O), and points 1, 2, 3, and 6 to cast iron (Fe, Si, and Mn).

**Figure 13:**
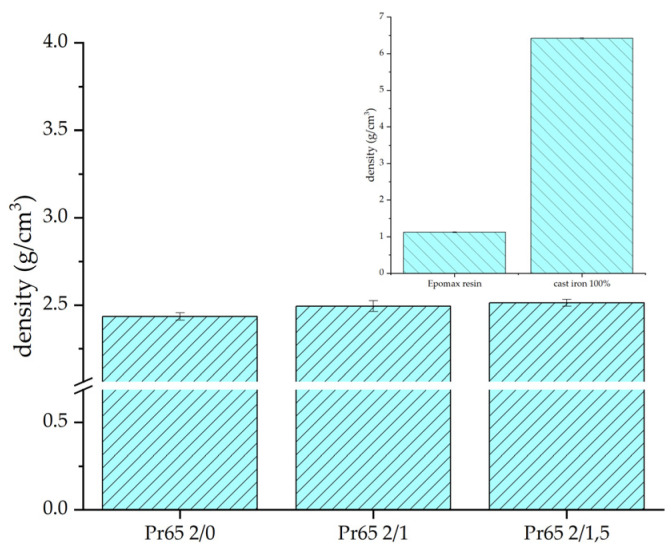
Density measurements of composites and reference samples.

Figure 13 shows the results of density measurements of GCI/epoxy-based composites and reference samples, i.e., epoxy resin and gray cast iron (window in the upper right corner). The determined density of the non-porous composite based on density measurements of reference samples of gray cast iron and epoxy resin was 2.508 g·cm^−3^. On this basis, the porosity of the composites produced in the tests was determined. The values of experimentally determined densities and porosities are listed in Table 3.

### 3.4. Mechanical Properties

#### 3.4.1. Static Tensile Test

**Figure 14:**
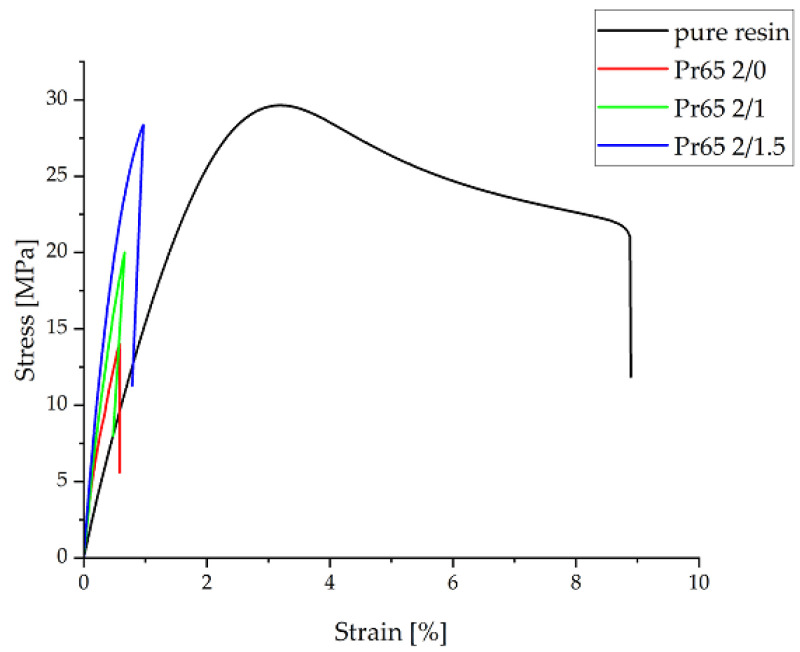
Graph of the stress–strain relationship under static tensile test conditions.

Figure 14 shows the relationship between the tensile stress, expressed in MPa, and the relative strain, expressed as a percentage. The curves presented in the chart show that GCI/epoxy-based composites have different properties compared to pure resin. These are brittle materials without a clear yield point. Table 4 summarizes the results obtained under static tensile test conditions for individual samples. Compared to pure epoxy resin, there was a significant increase in Young’s modulus and a decrease in relative deformation. The results indicate a beneficial effect of heating to a temperature of 130 °C and the heating time of the composite on the mechanical properties during a stable tensile test. The highest values of tensile strength and Young’s modulus were achieved by the Pr65 2/1.5 composite, which was heated for 90 min.

#### 3.4.2. Static Compression Test

**Figure 15:**
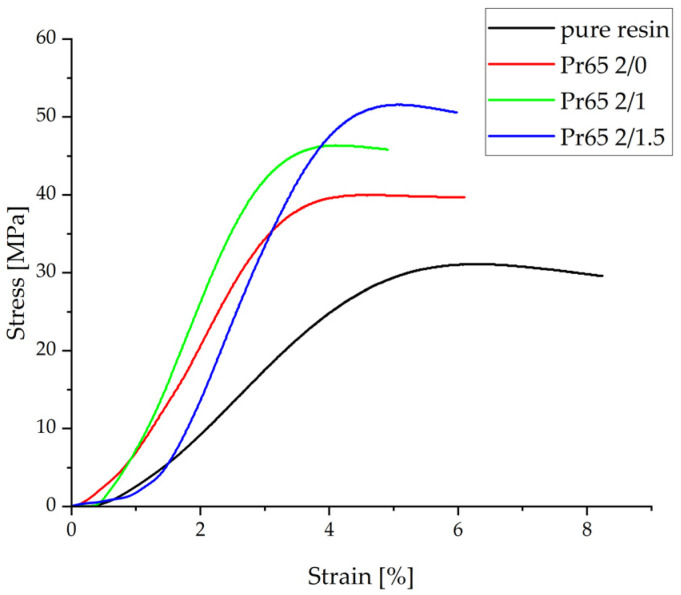
Graph of the stress–strain relationship under static compression tests.

A summary of the stress–strain relationship in the static compression test is presented in Figure 15. The obtained test results showed a significant increase in the compressive strength of the tested GCI/epoxy-based composites compared to pure epoxy resin (Table 5).

The curves and determined parameters indicate a beneficial effect of heating on the mechanical properties of composites under compression. The highest value of compressive strength and Young’s modulus was characteristic of the Pr65 2/1.5 composite, which was heated for 90 min at a temperature of 130 °C, while the lowest values of Young’s modulus and compressive strength were found for epoxy resin.

**Figure 16:**
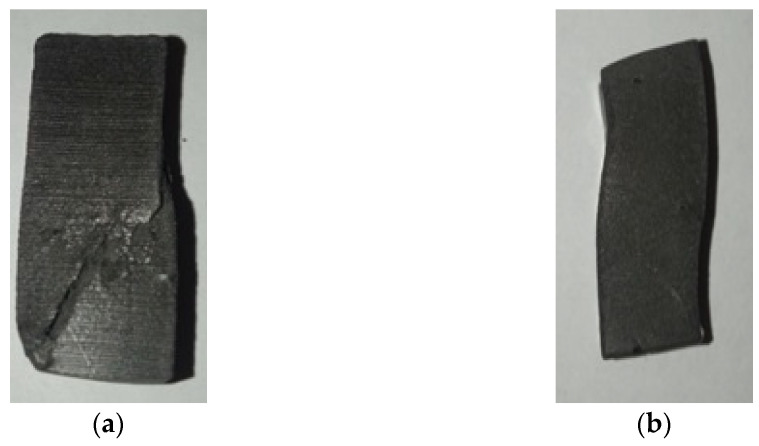
Photographs of samples after the static compression test of composites: (**a**) Pr65 2/0 and (**b**) Pr65 2/1.5.

Observations of the samples after static compressive strength tests revealed weak cohesion forces of the composites whose cross-linking process took place under RT conditions. Heating the GCI/epoxy-based composites at a temperature of 130 °C promoted cohesion forces in the composites of epoxy resin reinforced with gray cast-iron chips. For comparison, Figure 16 shows two samples after a static compression test of the composite not heated at 130 °C and the one heated at a temperature of 130 °C for 90 min.

The photo (Figure 16a) shows decohesion of the composite material not heated at 130 °C (Pr65 2/0) after a static compression test. The samples were destroyed when the maximum stress values for a given material were reached. In the case of the composite material heated at a temperature of 130 °C (Pr65 2/1.5), after removing the compressive force, the samples of these composites were not destroyed (Figure 16b), but returned to their initial dimensions.

#### 3.4.3. Static Flexural Test

**Figure 17:**
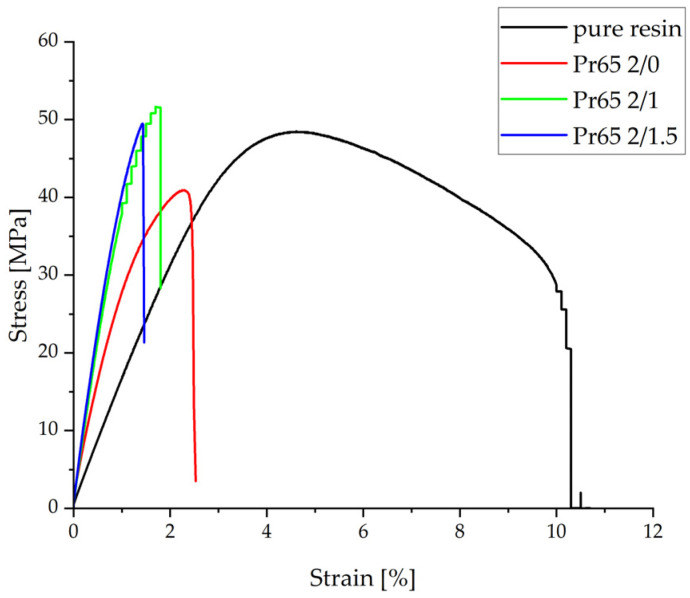
Graph of the flexural strength–displacement relationship under static flexural tests.

The test was performed as a single-point flexural test between two supports. The obtained results are presented in Table 6 and Figure 17.

As in the case of the static tensile test, significant differences were observed between the Young’s modulus values, and minor differences between the strength values of the epoxy resin and the GCI/epoxy-based composites. The Young’s modulus of the composite material compared to the modulus of pure epoxy resin was 1.8 to 2.7 times higher. The obtained results of static bending tests indicated that the composites are brittle materials. The curves shown in Figure 17 show a decrease in the deflection of composite samples with a simultaneous increase in the force acting on the sample. The highest value (Table 6) of strength in the static bending test was demonstrated by the Pr65 2/1.5 sample, similarly to the previous cases.

#### 3.4.4. Impact Strength

**Figure 18:**
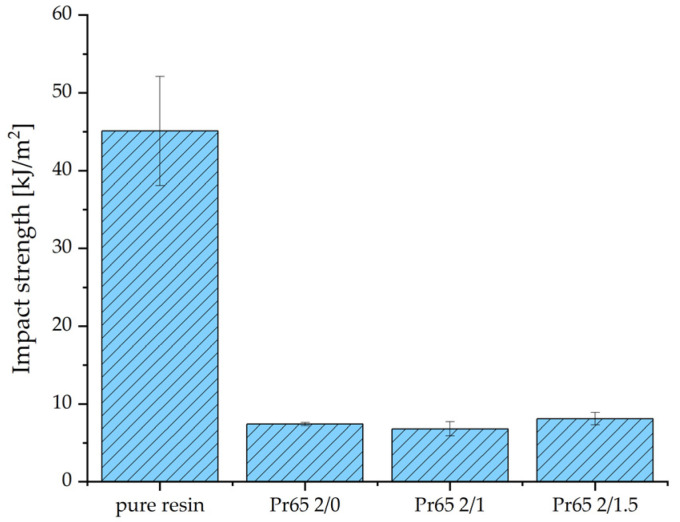
Composite Charpy impact test results.

The tests were carried out using a Charpy hammer with an energy of 2 J. Comparison of the results is presented in the chart in Figure 18. Cracking of the composite samples was observed at the boundaries of the connection between the cast-iron powder and the matrix. These observations indicate that the weakest elements of the GCI/epoxy-based composite structure are the interfacial interaction forces.

#### 3.4.5. Hardness Test

**Figure 19:**
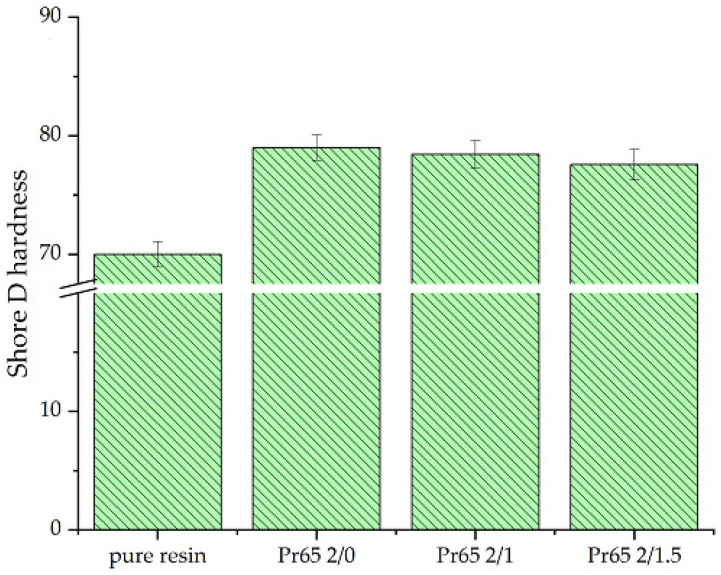
Comparison of the hardness values of composites and pure epoxy resin.

Figure 19 shows a comparison of the hardness values of the GCI/epoxy-based composites and pure epoxy resin. The hardness values of the composites were higher than the epoxy resin material. These values ranged from 75 to 80 on the Shore scale. The highest hardness value was characteristic of the composite not heated at 130 °C (Pr65 2/0), and the hardness values for the heated composites Pr65 2/1 and Pr65 2/1.5 were slightly lower. Differences in the hardness values of the composites were within the measurement uncertainties.

#### 3.4.6. Potentiodynamic Corrosion Testing

**Figure 20:**
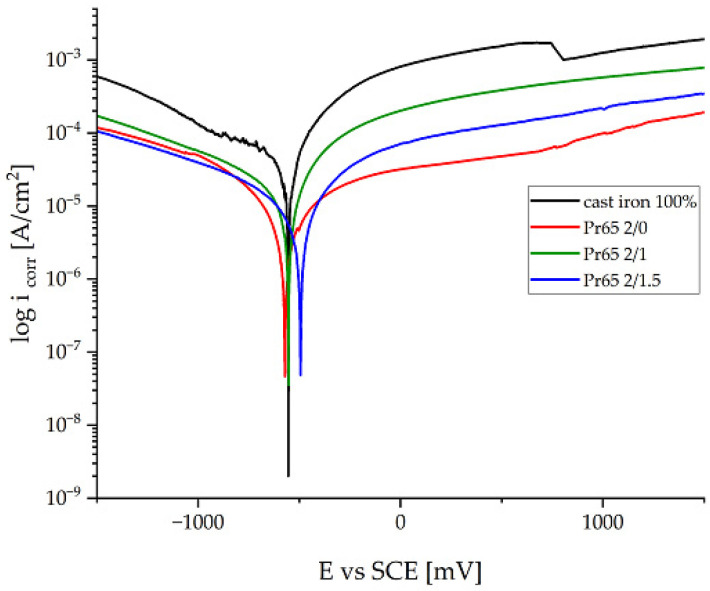
Comparison of anodic polarization curves of the composites and gray cast iron.

The comparison of the results of corrosion tests of the GCI/epoxy-based composites and the reference specimen of gray cast iron is shown in Figure 20. Table 7 summarizes the determined values of the corrosion parameters.

In the case of a gray cast-iron sample immersed in tap water, the corrosion potential was −567 V, and the corrosion current density reached 44.11 mA·cm^−2^ (Table 7). The values of the corrosion potential of the composites were slightly shifted towards higher values, compared to the reference material, which was gray cast iron, while the values of the corrosion current density were significantly lower (Figure 20). The corrosion potential of the Pr65 2/1.5 composite was shifted to higher values toward the anodic region. The Pr65 2/1.5 composite was characterized by the best corrosion resistance, measured by relatively higher resistance polarization values (R_pol_). The Pr65 2/1 and Pr65 2/0 composites showed less favorable corrosion properties compared to the Pr65 2/1.5 composite, but more favorable than gray cast-iron samples, as evidenced by lower corrosion current densities. The corrosion rate of gray cast iron was 3.3, 2.4, and 2.3 times higher than the corrosion rates of Pr65 2/1.5, Pr65 2/1, and Pr65 2/0 composites, respectively. Comparing the GCI/epoxy-based composites, it can be concluded that the soaking and longer heating time of the composites increased their resistance to electrochemical corrosion.

#### 3.4.7. Thermal Resistance of Composites

During thermal decomposition of epoxy resin, chemical bonds are broken and regenerated. Typically, thermal decomposition of any epoxy resin begins with dehydration of the secondary alcohol, which leads to the formation of vinylene ethers, followed by cleavage of ether or amine bonds. The amine bonds present in the cured epoxy resin show lower thermal stability than the ether bonds. Some of the remaining aromatic structures may undergo carbonization [48].

**Figure 21:**
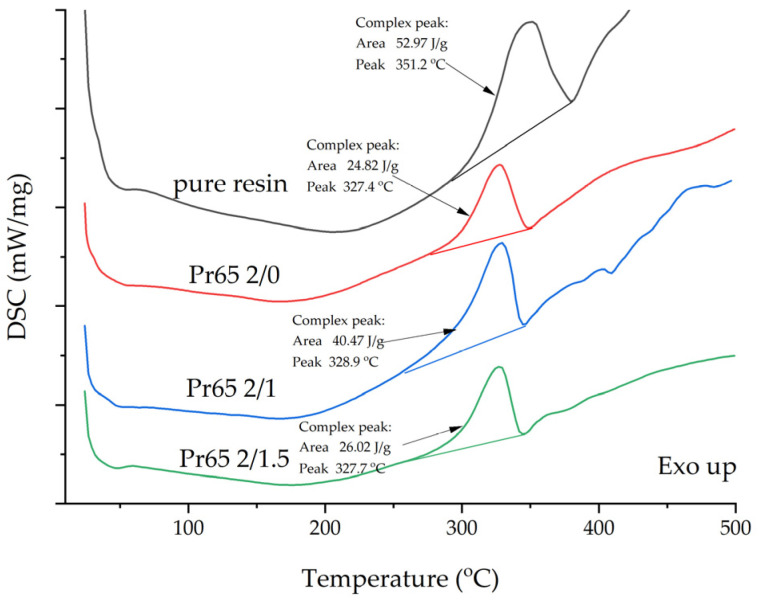
Comparison of the DSC curves of epoxy resin and composites decomposed under inert N_2_ gas atmosphere.

The results of measurements of the thermal decomposition of the GCI/epoxy-based composites and the reference sample, i.e., the specimen epoxy resin subjected to thermal decomposition in a nitrogen atmosphere, are shown in Figure 21. The area of the peak representing the internal thermal decomposition of the epoxy resin is a measure of the reaction energy [69]. The test results showed the highest resistance of epoxy resin compared to the composites. Its decomposition temperature was 351.2 °C and the exothermic peak area was 52.97 J/g. Despite minor differences between the composites, it should be noted that the best properties were demonstrated by the Pr65 2/1 composite, followed by Pr65 2/1.5 and Pr65 2/0 (Table 8). The addition of cast iron shifted the maximum of the exothermic peak by 22.3–23.8 degrees.

## 4. Conclusions

1. The matrix material was shown to be an epoxy resin based on bisphenol-A-diglycidyl ether (DGEBA)/bisphenol-A, and it is a key component of the investigated system. These results strongly suggested that epoxy resin should be regarded as a mixture of DGEBA and its oligomers, while the curing agent for epoxy resin is isophorone diamine (IPDA). An additional component of the system is benzyl alcohol.
2. The resin used was characterized by low viscosity, a relatively high content of volatile substances, and a noticeable concentration of epoxy groups.
3. The curing reaction of epoxy resin at 130 °C for the first 3 min was very fast. It took about 70 min to completely harden.
4. The epoxy resin had the highest tensile strength, and among the GCI/epoxy-based composites, the composites cured at 130 °C for 60 and 90 min had the highest tensile strength. The composites not heated at 130 °C had the lowest tensile strength value.
5. A longer heating time at 130 °C resulted in an increase in tensile strength and Young’s modulus. The lowest value of Young’s modulus was for the epoxy resin.
6. The composite heated at 130 °C for the longest time, i.e., 90 min, had the highest compressive strength, while the epoxy resin had the lowest. After removing the compressive force, the material of this composite returned to its original dimensions, while the composite not heated at a 130 °C was destroyed as a result of the static compression test.
7. The composite cured at 130 °C for 90 min had the highest value of strength in the static flexural test, while the composite not heated at a temperature of a 130 °C had the lowest. All composites were characterized by a higher value of Young’s modulus compared to the value of the modulus of pure epoxy resin.
8. GCI/epoxy-based composites had higher corrosion resistance compared to cast iron. The composite cured at a 130 °C for 90 min had the highest corrosion resistance. The soaking and longer heating time of the composites increased their resistance to electrochemical corrosion. The corrosion rate of cast iron under the measurement conditions was 3.3 times higher than the corrosion rate of the most resistant composite.
9. Epoxy resin and all GCI/epoxy-based composites showed high thermal resistance. Their thermal decomposition temperatures exceeded 300 °C. The addition of gray cast-iron powder to the epoxy resin shifted the maximum of the exothermic peak by more than 20 degrees. Epoxy resin was characterized by the highest thermal resistance. The difference between the decomposition temperatures of the composites did not exceed 1.5 degrees. Among the composites, the one cured at 130 °C for 60 min had the best properties, followed by the composite cured at 130 °C for 90 min. The composite not heated at a temperature of 130 °C had the lowest thermal resistance.
10. Comparison of the test results of the composites showed that the composite hardened at 130 °C for 90 min had the best mechanical properties. It was characterized by the highest tensile, compressive, and bending strength, the highest impact strength and corrosion resistance, and relatively good thermal resistance. Its tensile strength was similar to the tensile strength of epoxy resin.
11. Compared to gray cast iron, GCI/epoxy-based composites were characterized by a much lower weight and higher corrosion resistance. Compared to epoxy resin, composites reinforced with gray cast-iron powders were characterized by higher tensile, compressive, and flexural strength, as well as stiffness. These composites had low impact strengths due to their brittle and stiff behavior. The conducted research showed the possibility of a simple, highly efficient, and cheap method of utilizing of waste cast iron in the form of a powder.
12. The best mechanical and corrosion properties were characterized by the composite marked with the symbol Pr65 2/1.5, which was cured at a temperature of 130 °C for 90 min. This composite had a tensile strength of 28.35 MPa, comparable to epoxy resin, and a higher bending strength of 55.4 MPa and compressive strength of 53.8 MPa. This composite was thermally stable, its thermal decomposition temperature reached a value of 327.7 C, and in addition, in tap water, it showed a corrosion rate more than three times lower than cast iron used in practice for the production of water supply fittings.

## Figures and Tables

**Table 1:** Mechanical and strength properties with chemical composition of gray cast iron, GJL 250-2.

Tensile strength	(MPa)	268	C, mass% Si, mass% Mn, mass% P, mass% S, mass% Cr, mass% Fe, mass%	3.29–3.32 1.89–1.91 0.64–0.67 0.037–0.055 0.038–0.051 0.068–0.101 Remaining
Compressive strength	(MPa)	1064		
Flexural strength	(MPa)	400		
Young’s modulus	(GPa)	115		
Hardness	HB	196		

**Table 2:** Fundamental characteristics of epoxy resin.

Properties of Epoxy Resin	
Viscosity (η)	920 cP
Non-volatile-matter content (NV)	75.76%
Epoxy equivalent (EE)	192.38 g/mol

**Table 3:** Density and porosity of the GCI/epoxy-based composites.

Sample	Density g·cm^−3^	Porosity %
Pr65 2/0	2.450 ± 0.013	2.3
Pr65 2/1	2.488 ± 0.025	0.8
Pr65 2/1.5	2.500 ± 0.015	0.3

**Table 4:** List of strength parameters determined in the static tensile test.

Sample	Maximum Strength	Tensile Strength	Young’s Modulus	Relative Elongation
	N	MPa	GPa	%
Pr65 2/0	646 ± 20	14.8 ± 0,8	2.76 ± 0.07	0.59 ± 0.01
Pr65 2/1	882 ± 55	21.45 ± 1.45	3.82 ± 0.01	0.73 ± 0.06
Pr65 2/1.5	1115 ± 10	28.35 ± 0.25	4.51 ± 0.03	0.98 ± 0.15
Epoxy resin	1280 ± 70	28.7 ± 0.5	1.81 ± 0.04	8.9 ± 0.6

**Table 5:** List of strength parameters determined in the static compression test.

Sample	Maximum Strength	Compressive Strength	Young’s Modulus	Relative Elongation
	N	MPa	GPa	%
Pr65 2/0	4130 ± 7	39.6 ± 0.9	1.63 ± 0.07	4.62 ± 0.18
Pr65 2/1	5380 ± 20	49.2 ± 1.2	1.95 ± 0.16	4.27 ± 0.17
Pr65 2/1.5	5570 ± 15	53.8 ± 2.2	2.1 ± 0.1	5.00 ± 0.05
Epoxy resin	3480 ± 10	30.5 ± 1.1	0.77 ± 0.04	6.67 ± 0.17

**Table 6:** Strength properties obtained in the static flexural test.

Sample	Maximum Strength	Flexural Strength	Young’s Modulus	Displacement
	N	MPa	GPa	mm
Pr65 2/0	76.3 ± 3.3	40.4 ± 0.2	3.32 ± 0.07	2.45 ± 0.18
Pr65 2/1	89.5 ± 7.1	53.1 ± 1.4	4.78 ± 0.24	1.67 ± 0.05
Pr65 2/1.5	92.9 ± 2.9	55.4 ± 1.0	5.02 ± 0.01	1.51 ± 0.13
Epoxy resin	92.5 ± 16.4	50.8 ± 1.4	1.86 ± 0.12	9.72 ± 0.84

**Table 7:** Corrosion parameters of the composites exposed to tap water under RT conditions.

Samples	Corrosion Potential, E_corr_ (mV)	Corrosion Current Density, i_corr_ (mA·cm^−2^)	Anodic Tafel Coefficient, b_a_ (mV)	Cathodic Tafel Coefficient, b_c_ (mV)	Polarization Resistance, R_pol_ (Ω·cm^2^)	Corrosion Rate, CR (mm/year)
Pr65 2/0	−557	19.05	1154	2637	18,317	0.242
Pr65 2/1	−562	18.21	914	399	6639	0.231
Pr65 2/1.5	−497	13.21	1074	641	13,215	0.168
Cast iron 100%	−567	44.11	1014	340	2509	0.559

**Table 8:** Comparison of parameters characterizing thermal decomposition.

Sample	Thermal Decomposition Temperature, °C	Area of Exothermic Peaks, J/g
Epoxy resin	351.2	52.97
Pr65 2/0	327.4	24.28
Pr65 2/1	328.9	40.47
Pr65 2/1.5	327.7	26.02

## Data Availability

The original contributions presented in the study are included in the article, further inquiries can be directed to the corresponding author.
